# Spontaneous Cure of Consumption

**Published:** 1845-09

**Authors:** 


### SPONTANEOUS CURE OF CONSUMPTION.

No one who has read the authors on cousumption will doubt that spontaneous cures of the disease do occasionally take place. Perhaps this occurs oftener than is generally supposed; but there can be no doubt that much oftener, after a decided amendment, and when the friends of the patient have begun to flatter themselves that he is cured, the complaint returns with all its violence and carries its victim off. The educated physicain is at no loss how to account for these changes.

We lately saw a case of this illusive character. The husband of the lady, who had been the subject of consumption, assured us that she had been cured by brandy. A physician of unquestioned skill had pronounced her case one of genuine tubercular phthisis, and had pronounced the most unfavorable prognosis in reference to it. Indeed, the patient was worn down to such a degree that it was thought she would sink in a few days. At this stage she found herself with a strong propensity to drink brandy, and the appetite was indulged. She improved upon the regimen, and in a week or two was able to travel in a steamboat from one of our Southern cities to Louisville. She continued to improve after her arrival, and her husband was delighted at the thought of her perfect recovery. But was she cured? We were invited to see her; she had a cough, and complained of much soreness of the throat; her breathing was short, and her whole appearance anything but promising of health and long life. We made no minute examination, because we were invited to *see* the *cure* that had been made, and not to decide on the state of her lungs! It is such cures that, now and then, give temporary but unmerited credit to the various quack systems proposed for the remedy of this ruthless destroyer. Y.

